# Whipple’s disease presenting as weight gain and constipation in a Chinese woman

**DOI:** 10.1186/s12879-023-08276-y

**Published:** 2023-05-08

**Authors:** Haiyan Ye, Xiao Hu, Tommy Richard Sun-Wing Tong, Shuang Chen, Tao Li, Fanfan Xing, Jasper Fuk-Woo Chan, Kwok-Yung Yuen, Kelvin Hei-Yeung Chiu

**Affiliations:** 1 Department of Infectious Diseases and Microbiology, The University of Hong Kong-Shenzhen Hospital, Shenzhen, Guangdong China; 2 Department of Respiratory Medicine, The University of Hong Kong-Shenzhen Hospital, Shenzhen, Guangdong China; 3 Department of Pathology, The University of Hong Kong-Shenzhen Hospital, Shenzhen, Guangdong China; 4 Department of Radiology, The University of Hong Kong-Shenzhen Hospital, Shenzhen, Guangdong China; 5 Department of Microbiology, Queen Mary Hospital, Pokfulam, Hong Kong Special Administrative Region China; 6 State Key Laboratory of Emerging Infectious Diseases, Department of Microbiology, School of Clinical Medicine, Li Ka Shing Faculty of Medicine, Carol Yu Centre for Infection, The University of Hong Kong, Pokfulam, Hong Kong Special Administrative Region China

**Keywords:** Whipple’s disease, *Tropheryma whipplei*, Weight gain, IRIS

## Abstract

**Background:**

Whipple’s disease is a chronic infection due to *Tropheryma whipplei*, commonly reported in the Caucasian but not in the Chinese population.

**Case presentation:**

A 52-year-old female with good past health, was diagnosed with Whipple’s disease, presenting with constipation, unintentional weight gain, and fleeting polyarthralgia. Investigations prior to admission showed raised CA125 and computed tomography of the abdomen showed multiple retroperitoneal mesenteric lymphadenopathies. Extensive investigations performed on secondary causes of weight gain were unrevealing. Subsequent PET-CT scan revealed generalized lymphadenopathy involving the left deep cervical, supraclavicular, and retroperitoneal mesenteric area. Excisional biopsy of the left supraclavicular lymph node was performed, with histology showing infiltrations of Periodic acid-Schiff positive foamy macrophages. *T. whipplei* DNA was detected in her serum, saliva, stool, and lymph node by PCR targeting the 16S ribosomal RNA gene. She was started on intravenous ceftriaxone, and then stepped down to oral antibiotics for a total of 44 months. The recurrence of fever after 12 days of ceftriaxone raised the suspicion of Immune Reconstitution Inflammatory Syndrome (IRIS). Serial imaging showed a gradual reduction in the size of retroperitoneal lymphadenopathies. Literature review on Whipple’s disease in the Chinese population identified 13 reports of detectable *T. whipplei* DNA in clinical specimens. The majority of the cases were pneumonia, followed by culture-negative endocarditis, encephalitis, and skin and soft tissue infection. However, most patients with pneumonia were diagnosed based on next generation sequencing alone, with the resolution of pulmonary infiltrates without adequate duration of antibiotics, suggesting the possibility of colonization instead of infection. The recommendation of long-term doxycycline suppression after treatment may be supported by the slow response of retroperitoneal lymphadenopathies to antibiotics in our patient.

**Conclusions:**

Unintentional weight gain and constipation could be atypical presentations of Whipple’s disease. It is a rare disease in the Chinese population despite the advancement of molecular techniques in the diagnosis of infections. A prolonged course of antibiotics may be required due to slow clinical response as documented by serial imaging in our case. The possibility of IRIS should be considered in patients with breakthrough fever during treatment of Whipple’s disease.

## Introduction

Whipple’s disease is a chronic infection due to *Tropheryma whipplei*, first described by George H. Whipple in 1907 [1]. It is mostly reported in the Caucasian population, but only a few cases have been reported in the Asian population [2, 3]. Classical Whipple’s disease usually presents with polyarthralgia, chronic diarrhea, weight loss, and malabsorption. Here we report a case of Whipple’s disease in a Chinese middle-aged woman, with a rare presentation of unintentional weight gain, together with constipation, arthralgias, and peripheral and retroperitoneal lymphadenopathy.

## Case presentation

A 52-year-old female office worker at a security company had an incidental finding of elevated CA125 from 90 U/ml to 440 U/ml in one year during a routine check-up. She was then referred for further investigations to exclude abdominal and pelvic malignancies. Transvaginal ultrasonography showed uterine fibroids without ovarian abnormalities. Computed Tomography (CT) of the abdomen showed multiple retroperitoneal mesenteric lymphadenopathies. She enjoyed good past health, without the use of any over-the-counter medication. She has no personal history or contact history of pulmonary tuberculosis. She kept no pets at home. She was a non-smoker and a social drinker. She denied any recent travel history. She was married and lived with her husband. She enjoyed consumption of raw salmon and sushi once per week for the last 5 years.

She was then admitted to our hospital in July 2018 for further investigation of retroperitoneal lymphadenopathy. Further history taking revealed constipation and an unintentional weight gain of 7.5 kg in the previous two years. She volunteered malaise, poor memory, and insomnia in the recent few months. She experienced frequent attacks of migratory arthralgia involving small fingers and occasional shoulder and ankle joints with spontaneous resolution within 2 weeks. She denied any diarrhea, abdominal pain, and other symptoms of systemic illness. The patient was obese based on the Asia-Pacific Standard of BMI, with a body weight of 70 kg, and BMI 28 kg/m^2^. There were no palpable lymphadenopathies, rash, or malformation of the extremities. Abdominal, respiratory, cardiovascular, and neurological examinations were all unremarkable.

## Investigations

Laboratory examinations revealed normal peripheral blood count, elevated Erythrocyte sedimentation rate (ESR) (65 mm/h), C reactive protein (CRP) (68 mg/L), and CA 125 (440 U/ml). Autoimmune markers including rheumatoid factor, antinuclear antibody (ANA), anti-double stranded DNA (anti-dsDNA), and Antineutrophil Cytoplasmic Antibodies (ANCA) were all negative. Serum C3, C4, IgG, IgM, and IgA were within normal range. Brucella antibody, human immunodeficiency virus antibody and antigen tests were negative. In view of the unexplained weight gain in recent 2 years, thyroid function test, HbA1c, morning cortisol and paired ACTH were performed and were within normal range.

**Fig. 1:**
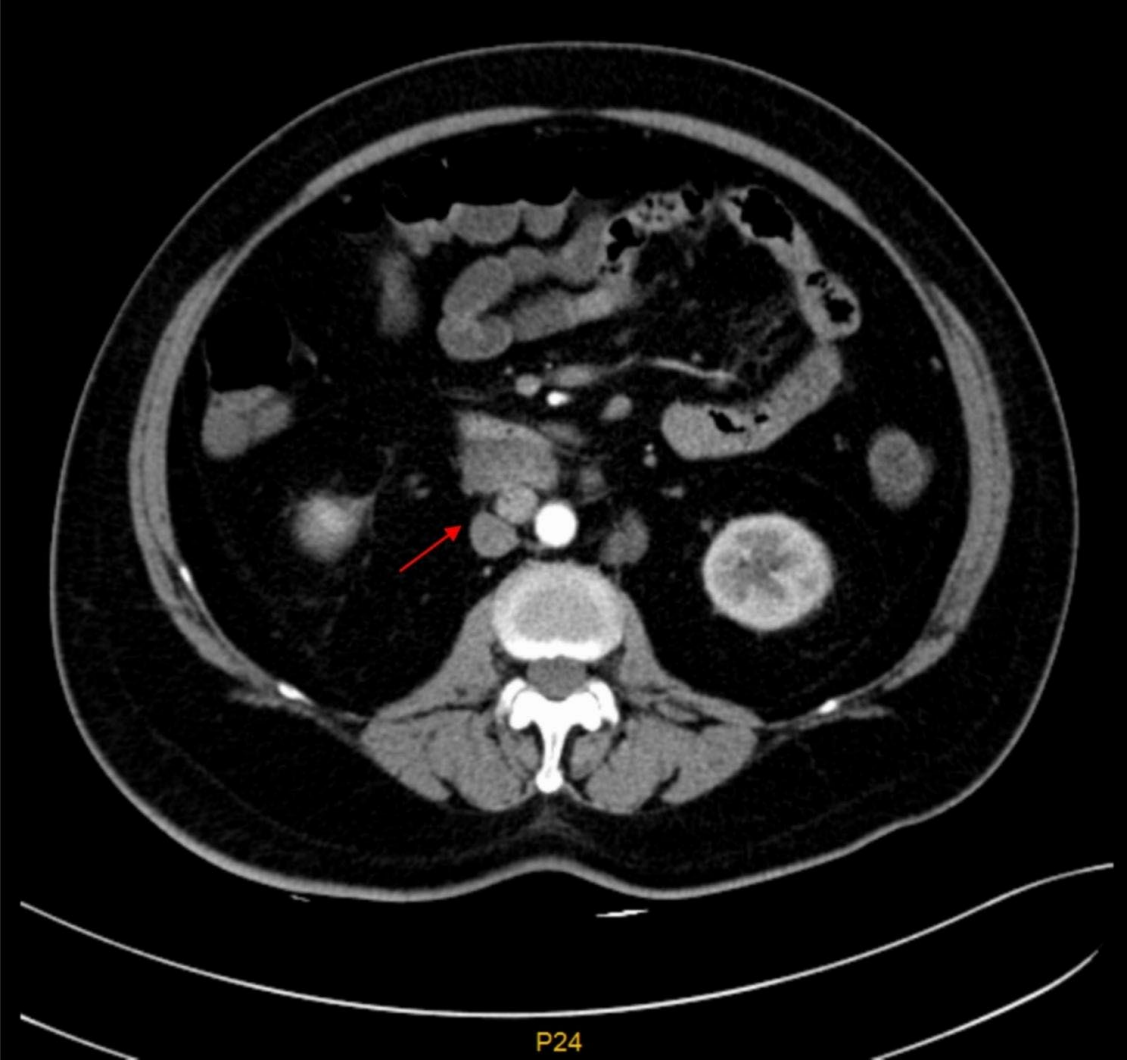
CT abdomen showing multiple retroperitoneal lymphadenopathies. The arrow points to the largest conglomerate of lymphadenopathies (arrow)

**Fig. 2:**
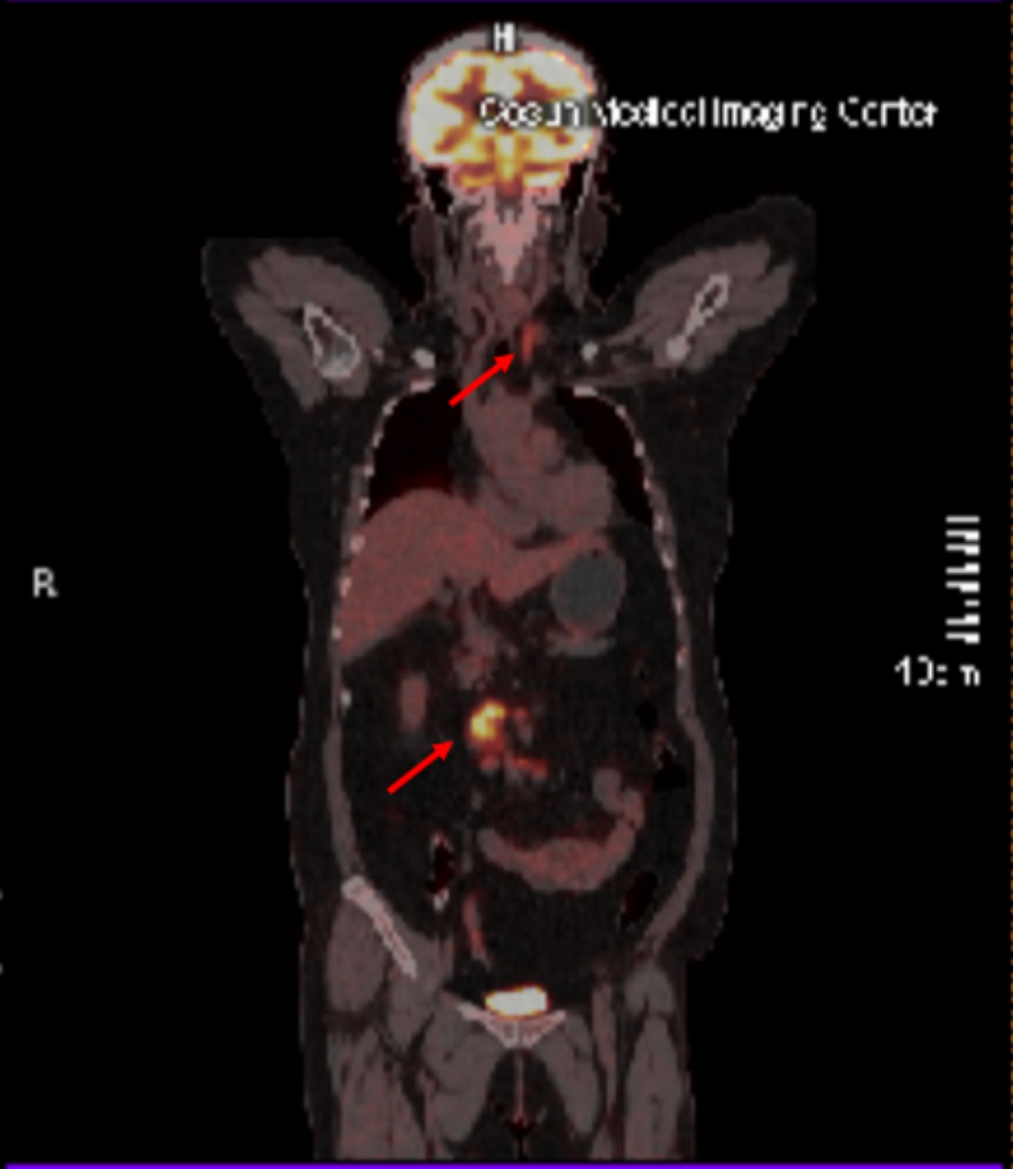
PET-CT scan showing left cervical and supraclavicular lymphadenopathy with retroperitoneal lymphadenopathy (arrows)

Further endoscopic examinations were performed in view of raised CA125. Oesophago-gastro-duodenoscopy showed chronic non-atrophic gastritis and gastric polyp with subsequent biopsy confirmed to be benign fundic gland polyp. Colonoscopy was also unremarkable. Chest X-ray showed a normal cardiothoracic ratio. CT Abdomen (Fig. 1) showed multiple retroperitoneal mesenteric lymph nodes, and the largest lymph node was 32.0 × 17.2 mm in size. Further Positron Emission Tomography-Computed Tomography (PET-CT) scan revealed generalized lymphadenopathy involving the left deep cervical, supraclavicular, and retroperitoneal mesenteric area (Fig. 2).

**Fig. 3:**
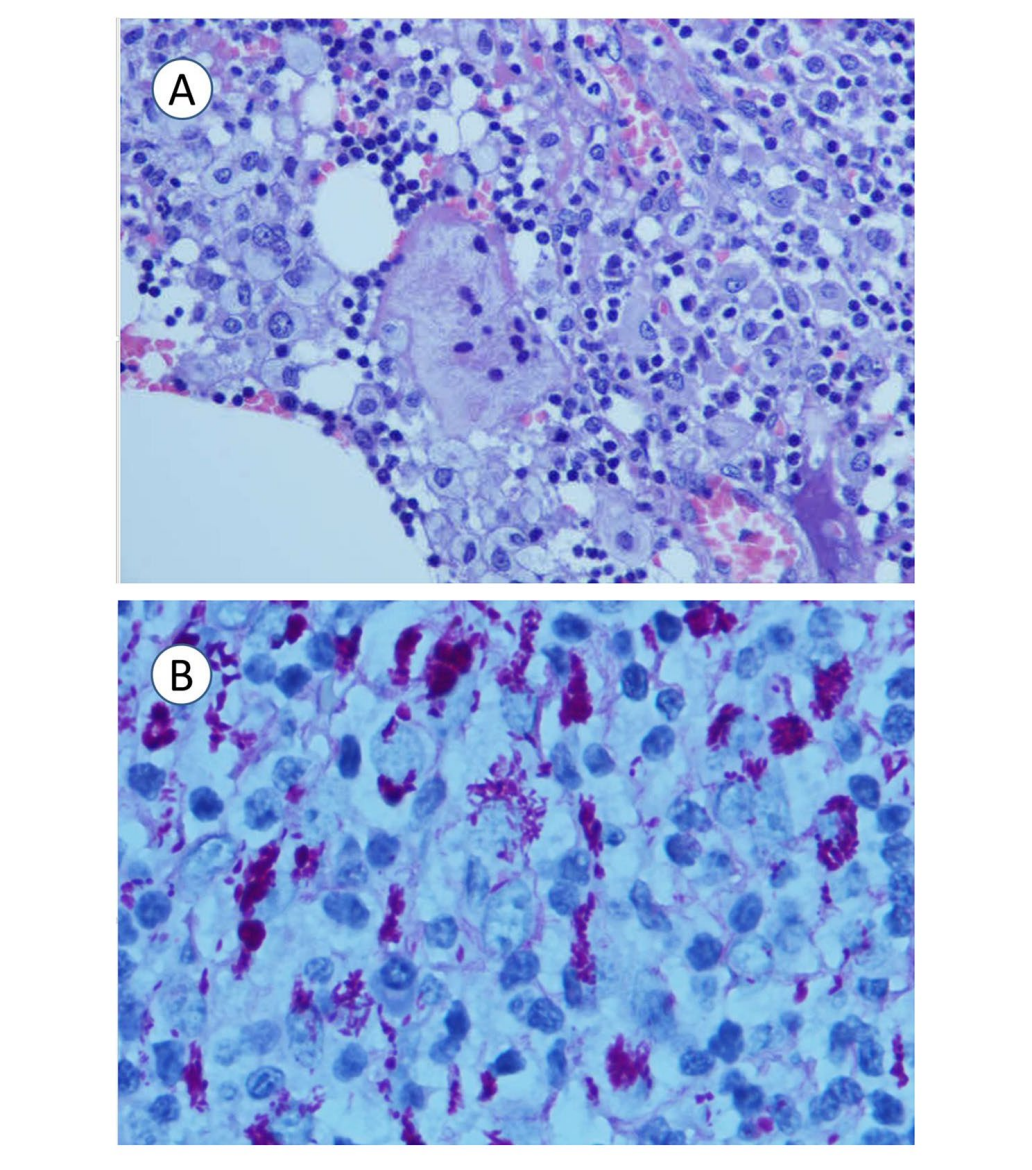
A: Histology of the lymph node showed massive infiltration of the lymph node by foamy macrophages by H&E stain (10 × 40). B: Periodic acid-Schiff (PAS) staining with PAS-positive macrophages (10 × 100)

**Fig. 4:**
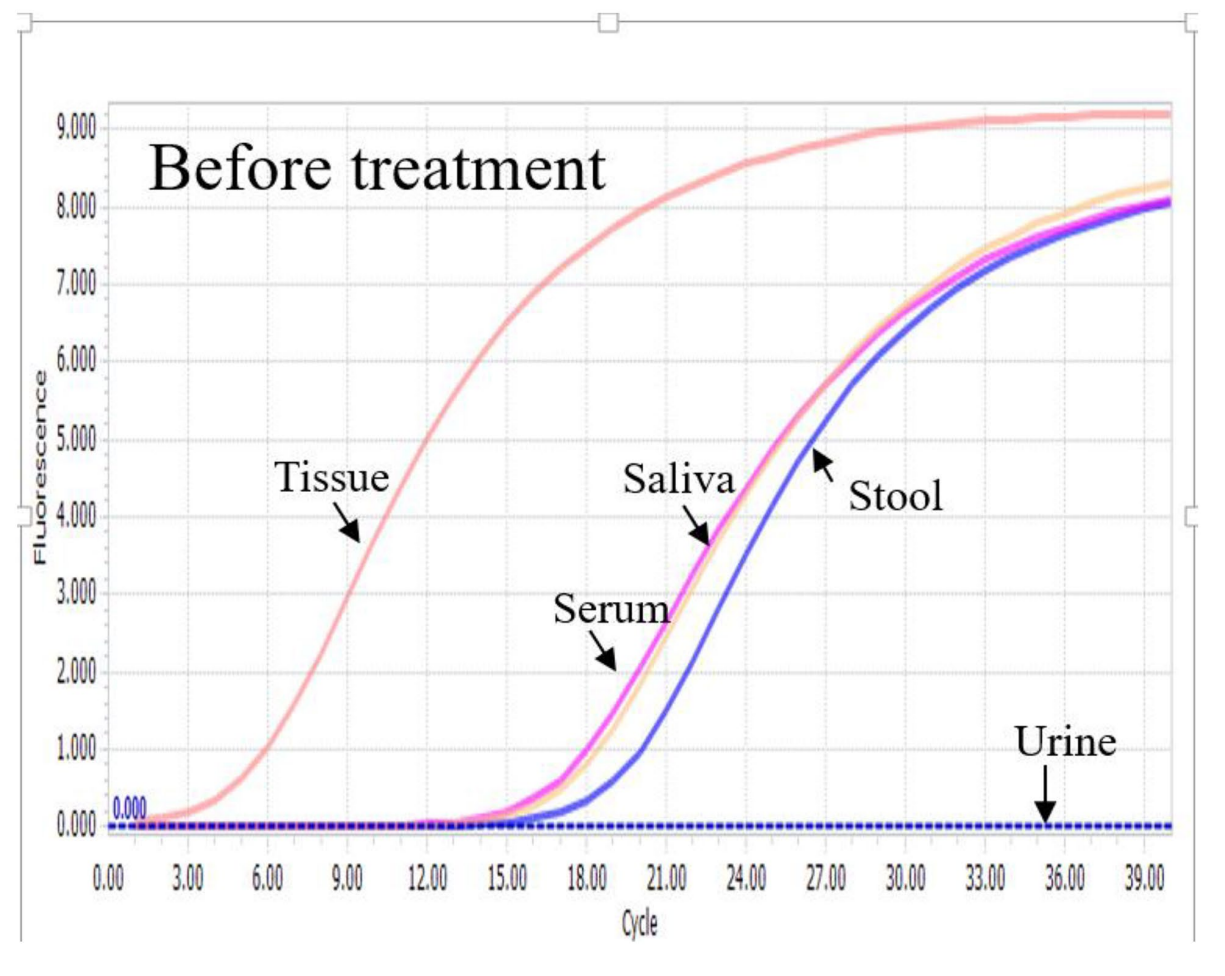
PCR amplification curve of *Tropheryma whipplei* real-time PCR on different clinical specimens of our patient

Excisional biopsy of the left supraclavicular lymph node was performed. Microbiological investigations on the lymph node showed growth of coagulase-negative Staphylococcus from bacterial culture but negative fungal and mycobacterial culture. *Mycobacterium tuberculosis* DNA was not detected by PCR. The histology of the lymph node showed massive infiltration of foamy macrophages by hematoxylin and eosin stain (Fig. 3). There was no evidence of granulomatous inflammation and malignancy. However, on further Periodic acid-Schiff staining, numerous PAS-positive macrophages were found, raising suspicion of Whipple’s disease. Subsequent *T. whipplei* real-time PCR using the 16S ribosomal RNA gene was performed, with *T. whipplei* DNA detected in the patient’s serum, saliva, stool, and the left supraclavicular lymph node, but not in the urine (Fig. 4). Further sequencing of the PCR products showed 100% nucleotide identity to the reference genome of *T. whipplei*. The diagnosis of Whipple’s disease was made based on the typical histopathology and detection of *T. whipplei* DNA in clinical specimens. Further investigations were performed to rule out the involvement of other organ systems. Ophthalmological examination revealed no evidence of uveitis and chorioretinitis. Transthoracic echocardiography showed no vegetation. Magnetic resonance imaging of the brain with contrast was unremarkable.

## Treatment

The patient was started on intravenous ceftriaxone with an improvement of joint symptoms on the second day of antibiotics administration. However, the patient redeveloped fever of 39.4℃, sore throat as well as an erythematous lesion over the lips after 12 days of treatment. CRP was elevated from 68 to 162 mg/L, ESR was elevated from 65 to 90 mm/h. Nasal swab for respiratory virus antigen and stool for enterovirus PCR were negative. Serum and stool for *T. whipplei* specific PCR were negative. The fever subsided after 2 days without intervention along with a decrease in inflammatory markers. She was then switched to Doxycycline 100 mg twice daily and hydroxychloroquine 200 mg three times daily for maintenance therapy.

## Outcome and follow up

**Fig. 5:**
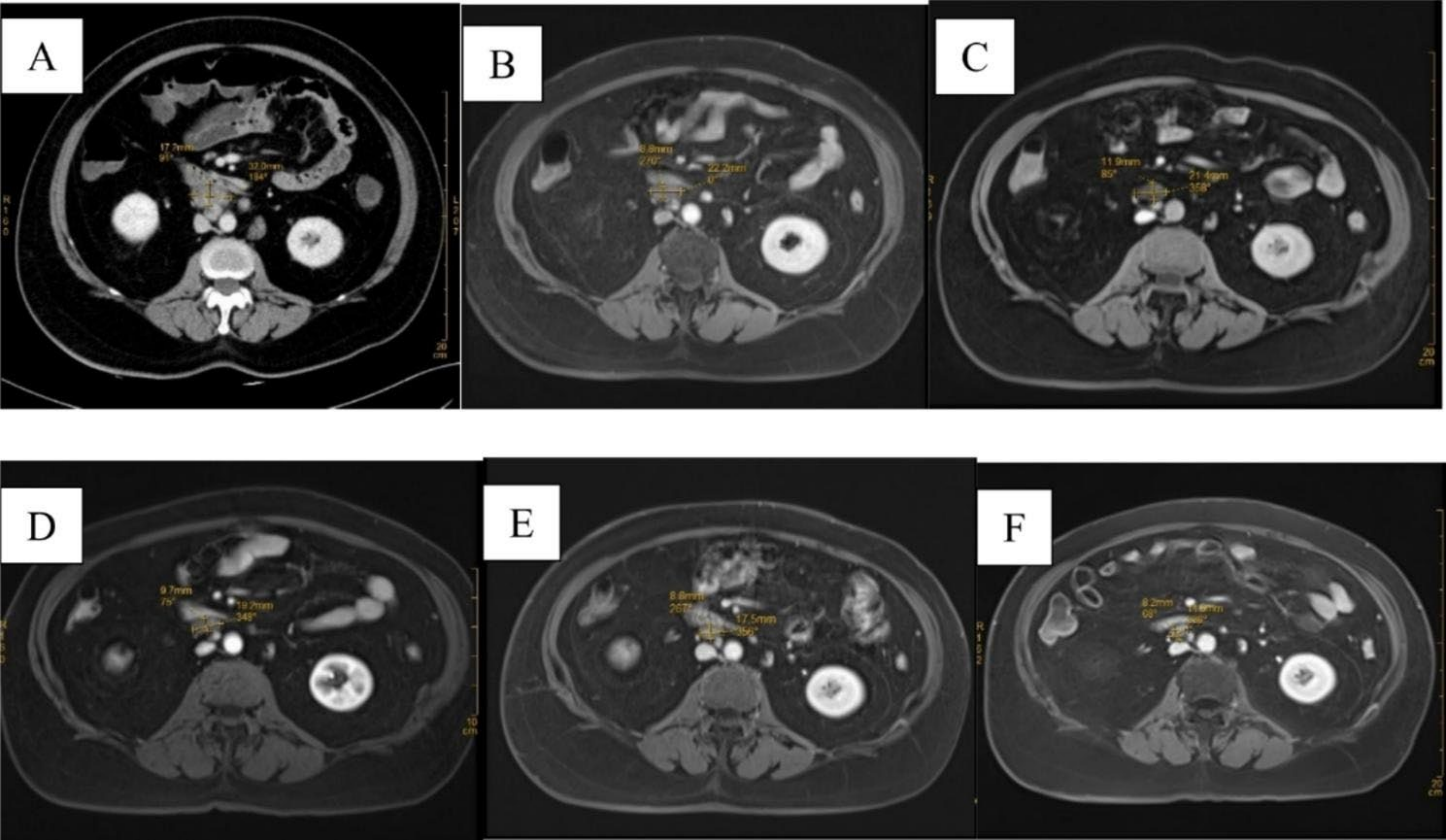
Serial Imaging of abdomen; A: CT abdomen showed the size of the largest retroperitoneal lymph node was 17.2 × 32.0 mm on July 2018 (Before treatment); B: MRI abdomen showed the size of the largest retroperitoneal lymph node was 8.2 × 22.2 mm on November 2018 (After 4 months of Doxycycline and Hydroxychloroquine); C: MRI abdomen showed the size of the largest retroperitoneal lymph node was 11.9 × 21.4 mm on July 2019 (After 6 months of trimethoprim/sulfamethoxazole); D: MRI abdomen showed the size of the largest retroperitoneal lymph node was 9.7 × 19.2 mm on January 2020 (After 12 months of trimethoprim/sulfamethoxazole); E: MRI abdomen showed the size of the largest retroperitoneal lymph node was 8.8 × 17.5 mm on July 2021(After 18 months of Doxycycline and Hydroxychloroquine); F: MRI abdomen showed the size of the largest retroperitoneal lymph node was 8.2 × 14.6 mm on May 2022 (After 28 months of Doxycycline and Hydroxychloroquine)

After 1 month of antimicrobial treatment, the patient demonstrated marked improvement in terms of blood parameters and clinical symptoms. Blood parameters including CRP, ESR, and CA125 gradually improved. There were no recurrences of constipation and arthralgia. Her body weight gradually decreased from 70 kg to 61 kg in one year. She was switched from doxycycline and hydroxychloroquine to trimethoprim/sulfamethoxazole 960 mg twice a day due to the development of transient blurring of vision after five months of treatment. However, she was later switched back to doxycycline and hydroxychloroquine combination due to suboptimal improvement of the size of lymphadenopathy in serial imaging. Serial cross-sectional images were shown in Fig. 5, showing a gradual reduction in size from 17.2 × 32.0 mm to 8.2 × 14.6 mm after a total of 44 months of treatment. Therefore, the patient decided to stop treatment and she did not develop any relapse of symptoms 6 months after stopping antibiotics.

## Literature review

Classical Whipple’s disease typically affects Caucasian populations, but it is seldom reported in the Asian population. In order to understand the clinical characteristics of this disease in the Chinese population, a literature review was performed using the search terms ‘Whipple’s disease’, ‘*Tropheryma whipplei*’, and ‘China’ in PubMed. Only articles in English were included in this review.

## Results

Thirteen reports of detectable *T. whipplei* DNA in clinical specimens, reported from 2007 to 2022, were identified in the search [4–11]. Clinical details were summarized in Table 1. The majority of patients were male (9 out of 13 patients (69.2%)), with ages ranging from 23 to 81 years old (median age 44 years old). Four patients were immunocompromised due to underlying diabetes mellitus, dermatomyositis, and acquired immunodeficiency syndrome (AIDS). The majority of the cases reported was pneumonia, followed by culture-negative endocarditis, encephalitis, and skin and soft tissue infection. All cases of pneumonia were diagnosed by next generation sequencing or nanopore sequencing on bronchoalveolar lavage alone without histology support. Other cases of extrapulmonary infection were diagnosed by demonstration of Periodic acid-Schiff (PAS) stain positive macrophages in biopsy with *T. whipplei* DNA detected by PCR. For patients with underlying pneumonia, three of the patients had other co-existing pathogens to explain the development of pulmonary infiltrates including *Acinetobacter baumannii*, *Pneumocystis jirovecii*, and *Mycobacterium tuberculosis*. Furthermore, three other patients had underlying interstitial lung disease, which may also explain the development of pulmonary deterioration of the patient. The majority of the patients with pneumonia responded clinically without the administration or maintenance of effective treatment against *T. whipplei*.

**Table 1:** Characteristics of the thirteen patients with *T. whipplei* infection in China

Year	Age/sex	Presentation	Organ involvement	Diagnosis method	Comorbidities	Treatment	Outcome
2007 [4]	30/M	Transient loss of consciousness, hypologia, alexia	Brain	PAS(+) PCR	Nil	Amoxicillin, and chloromycin	Nil
2017 [5]	Case 1: 54/F	Nil	Mitral valve	PAS(+) PCR	Nil	Nil	Nil
	Case 2: 64/F	Nil	Left atrium, mitral valve	PAS(+) PCR	Nil	Nil	Nil
2021 [6]	Case 1: 39/F	Cough, shortness of breath, fever	Lung	mNGS of BAL	Nil	Sulfamethoxazole, meropenem and fluconazole for 17 days	Recovered
	Case 2: 81/M	Cough, shortness of breath	Lung	mNGS of BAL	Nil	Sulfamethoxazole, voriconazole and methylprednisolone for 5 days	Death
2021 [7]	23/F	Respiratory distress, shortness of breath, fever, weight loss, joint pain	Lung	mNGS of BAL	Nil	Imipenem, fluconazole, polymyxin B for 18 days (For treatment of carbapenem-resistant *Acinetobacter baumannii* pneumonia)	Recovered
2021 [8]	28/M	Dry cough, shortness of breath	Lung	mNGS of BAL	AIDS	Sulfamethoxazole-trimethoprim (For treatment of *Pneumocystis jirovecii* infection), Meropenem, caspofungin, methylprednisolone for 16 days	Recovered
2021 [9]	44/M	Dry cough, shortness of breath	Lung	mNGS of BAL	DM, HCV infection	Trimethoprim/sulfamethoxazole, isoniazid, rifapentine, amikacin, ethambutol (For treatment of *Mycobacterium tuberculosis*)	Recovered
2021 [10]	Case 1: 64/M	Cough and dyspnea	Lung	Nanopore of BAL	DM, Interstitial lung disease	Streptomycin and penicillin G for 2 weeks, then trimethoprim/sulfamethoxazole	Recovered
	Case 2: 31/M	Fever, cough, intermittent diarrhea, weight loss, and arthralgia	Lung	Nanopore of BAL	Dermatomyositis, Interstitial lung disease	Voriconazole, levofloxacin, ganciclovir, sulfamethoxazole (duration not mentioned)	Recovered
	Case 3: 59/M	Dry cough	Lung	Nanopore of BAL	Interstitial lung disease	None	Nil
2021 [11]	26/M	Left chest pain	Lung	mNGS of BAL	Nil	Ceftriaxone for 2 weeks, then trimethoprim/sulfamethoxazole (duration not mentioned)	Recovered
2022 [12]	62/M	Multiple subcutaneous nodules	Skin	PAS(+) PCR	Nil	Doxycycline, and hydroxychloroquine for 1 year	Recovered

AIDS = Acquired immunodeficiency syndrome; BAL = Bronchoalveolar lavage; DM = Diabetes mellitus; HCV = Hepatitis C virus; mNGS = metagenomics next generation sequencing; PAS = Periodic acid-Schiff; PCR = Polymerase chain reaction

## Discussion and conclusion

Whipple’s disease is a rare multisystemic infection caused by the Gram-positive bacillus *T. whipplei*, which is ubiquitous in the environment. It was previously reported that *T. whipplei* can be detected in sewage and it is more commonly found in the fecal samples of sewage workers (12 to 26%) than that of the general population [13, 14]. Not all individuals develop Whipple’s disease upon exposure to *T. whipplei*. Patients with classical Whipple’s disease are typically Caucasian males with occasional exposure to soil or animals, and usually present with arthralgia/ arthritis, diarrhea, and weight loss [15]. Our patient, however, is a Chinese woman presenting with arthralgia, and generalized lymphadenopathy, together with weight gain instead of weight loss. Extensive investigations have been performed to rule out secondary causes of unintentional weight gain, including thyroid function test to rule out hypothyroidism, morning cortisol and ACTH to rule out Cushing’s syndrome, CXR and ECG to rule out congestive heart failure, together with systemic imaging including CT and PET-CT to rule out other pathology that may explain for the atypical presentation of the disease. Furthermore, with the initiation of treatment, the body weight of the patient gradually returned to normal without any intentional weight loss, suggesting that weight gain together with constipation could be atypical presentations of Whipple’s disease. There are other case reports in the literature describing patients with Whipple’s disease presenting with weight gain, with body weight returning to baseline after successful antibiotic treatment [16].

The diagnosis of Whipple’s disease is usually made by demonstration of PAS-positive macrophages or a positive PCR from clinically affected tissues such as duodenal biopsy [17]. Immunohistochemical staining has also been used in some centers on tissue specimens for better visualization of the organisms [18], however, the sensitivity is usually lower when compared with PCR. Although *T. whipplei* was first successfully isolated from mammalian cell cultures in 2000 [19], culturing *T. whipplei* requires expertise and it is labour intensive, and the technique for performing culture is not available in most microbiology laboratories. Serological assays have been established to identify patients with Whipple’s disease [20], but the test was only performed in a small study; therefore, further validation is required to validate its specificity and sensitivity.

Our literature review revealed the majority of the cases reported in China were diagnosed by the detection of *T. whipplei* DNA by next generation or nanopore sequencing alone on the bronchoalveolar lavage. However, this raised suspicion of whether these cases should be considered Whipple’s disease. Firstly, not all patients with *T. whipplei* will develop Whipple’s disease. Previous studies have demonstrated the detection of *T. whipplei* DNA by PCR on saliva in asymptomatic patients [13]. One study suggested that *T. whipplei* can be an oral commensal in one-third of healthy individuals [21]. Furthermore, most of the patients reported had alternative explanations for the development of pulmonary infiltrates, such as co-existing infection or underlying interstitial lung disease. In addition, most patients responded clinically without the initiation of long-duration of antibiotics. Therefore, these cases may simply be incidental findings of colonization of *T. whipplei* instead of Whipple’s disease.

Whipple’s disease can be fatal without treatment with antibiotics. Commonly chosen antibiotics include doxycycline, ceftriaxone, meropenem, and rarely a combination of chloroquine and minocycline. Trimethoprim/sulfamethoxazole is recommended as an alternative in some papers [22, 23], but in vitro tests have confirmed that trimethoprim is not active, and late relapses with resistance to sulfamethoxazole have been described [24]. There were also reports of treatment failure and relapses in all of the 14 classic Whipple’s disease patients receiving trimethoprim/sulfamethoxazole alone, suggesting it is not an optimal treatment for Whipple’s disease. Concerning the duration of antibiotics, some researchers [25] recommend initial treatment with intravenous antibiotics for two weeks to achieve good penetration across the blood-brain barrier, followed by oral doxycycline and hydroxychloroquine for a year, then stepped down to long-term suppression with doxycycline due to previous reports of fatal relapses after 1 year of treatment [26]. This could be supported by our case report that with serial cross-sectional imaging of the abdomen, despite a prolonged course of antibiotics, there is still residual lymphadenopathy seen in the retroperitoneal area, hence suggesting there may be a need for serial monitoring of the involved area of Whipple’s disease so as to decide the exact duration of antibiotics. Patients should also be warned about the chance of relapse after cessation of antimicrobials.

IRIS has been clinically defined as a paradoxical flare-up of inflammatory signs and symptoms beginning after the initiation of effective antimicrobial treatment. During antimicrobial treatment, up to 10% of classic Whipple’s disease patients are affected by IRIS [27]. However, a definitive laboratory test for IRIS is lacking. The diagnosis relies largely on clinical judgment, and various research groups have proposed slightly different definitions for IRIS [28]. Previous immunosuppressive therapy and low CD4 + cell count before initiation of antimicrobial treatment were considered risk factors of IRIS in patients with Whipple’s disease [29]. Furthermore, new research showed that intestinal barrier dysfunction and microbial translocation in Whipple’s disease patients were highly predictive for the onset of IRIS [30]. Although all investigations were unremarkable after the development of fever after the initiation of effective antibiotics, it is uncertain whether such manifestation represents IRIS. The clinical manifestations of our patient do not meet all the diagnostic criteria of IRIS from the previous cohort study, and she did not have the risk factors of IRIS mentioned above. However, the resolution of fever without immunosuppressive therapy did not exclude IRIS, as not all patients with IRIS necessitate the use of immunosuppressants in the literature [31]. Further studies on Whipple’s disease in the Chinese population are required to understand the atypical presentation of this rare disease.

In conclusion, unintentional weight gain and constipation could be atypical presentations of Whipple’s disease. It is a rare disease in the Chinese population despite the advancement of molecular techniques in the diagnosis of infections. A prolonged course of antibiotics may be required due to slow clinical response as documented by serial imaging in our case. The possibility of IRIS should be considered in patients with breakthrough fever while on the correct antibiotics for Whipple’s disease.

## Data Availability

The data are available from the corresponding author upon reasonable request.

## Abbreviations

ANA: Antinuclear antibody
anti-dsDNA: anti-double stranded DNA
ANCA: Antineutrophil Cytoplasmic Antibodies
ACTH: Adrenocorticotropic Hormone
AIDS: Acquired immunodeficiency syndrome
CT: Computed Tomography
CRP: C-reactive protein
ESR: Erythrocyte sedimentation rate
IRIS: Immune Reconstitution Inflammatory Syndrome
*T. whipplei*: *Tropheryma whipplei*
PET-CT: Positron Emission Tomography-Computed Tomography
PAS: Periodic acid-Schiff
PCR: Polymerase chain reaction
